# Morbidity and mortality from ingested button batteries higher than generally known

**DOI:** 10.1007/s00405-025-09690-5

**Published:** 2025-10-24

**Authors:** Frederik G. Dikkers, Tjark Ebels

**Affiliations:** 1 Department of Otorhinolaryngology/Head and Neck Surgery, Amsterdam UMC location Vrije Universiteit Amsterdam, De Boelelaan 1117, Amsterdam, The Netherlands; 2 University Medical Center Groningen, department of Otorhinolaryngology/Head and Neck Surgery, University of Groningen, Hanzeplein 1, Groningen, 9713 GZ The Netherlands; 3 University Medical Center Groningen, department of Cardiothoracic Surgery, University of Groningen, Hanzeplein 1, Groningen, 9713 GZ The Netherlands

The number of children worldwide who die or suffer serious complications each year as a result of ingesting button battery is unknown. However, with the limited information available, it is possible to calculate the likely size of this annual number of unfortunate, otherwise healthy, children worldwide.

The term “button battery” (BB), as used in this article, includes coin cells, button cell batteries, and coin batteries. Once a BB becomes lodged in a body cavity - usually the hypopharynx or esophagus - an immediate electrical short circuit is created by ambient electrolytes. If a target device powered by a BB no longer works to expectations, the common supposition is that the BB is empty. However, the BB still has considerable residual voltage. Therefore, an electrical short circuit can be caused by both new and “empty” batteries, and their damaging effect on body tissue is similar. The short circuit initiates hydrolysis of water into hydrogen gas and hydroxide ions, resulting in a pH of 12 or higher at the negative pole. Subsequently, the continued combination of progressive colliquation necrosis results in tissue degradation and loss. This process can have serious consequences at any location where the BB accidentally becomes lodged, especially the hypopharynx, esophagus, trachea, larynx and any other body orifice [1, 2]. In addition to becoming lodged in easily accessible cavities in the head and neck area, a BB can also become lodged in the eye [3], vagina [4], or even under a plaster cast [5].

The estimated global number of victims depends on several factors. The first and most important factor is underreporting. According to a 2022 survey of more than 400 physicians who directly manage BB ingestions [6], only 11% of BB injuries and 4% of all foreign body ingestions or aspiration cases were reported to a data source. Second, the number of children per million population varies between countries. Third, the quality of national health care varies from country to country. Fourth, the number of BBs sold is neither freely nor publicly available. It is claimed that it will exceed 4.5 billion units in 2024, and is expected to exceed 6.7 billion units by 2031 [7]. Most BBs are 20 mm in diameter (usually coded CR20xx, where CR stands for lithium and xx is the thickness in tenths of mm). This is precisely the size most likely to become lodged in the pediatric esophagus.

Estimates of the number of victims do not exist, due to the lack of effective and comprehensive registries. In the US, there was a voluntary registry formerly maintained by the National Capital Poison Center, a non-governmental organization (www.poison.org). It stopped registering national figures on button battery ingestion after 2019. Since then, there is a National Battery Ingestion Hotline (www.batteryingestionhotline.com). However, their recorded number of ingestions is much lower than the latest figures from the National Capital Poison Center. A comprehensive comparison between the two registries has not been published.

**Fig. 1:**
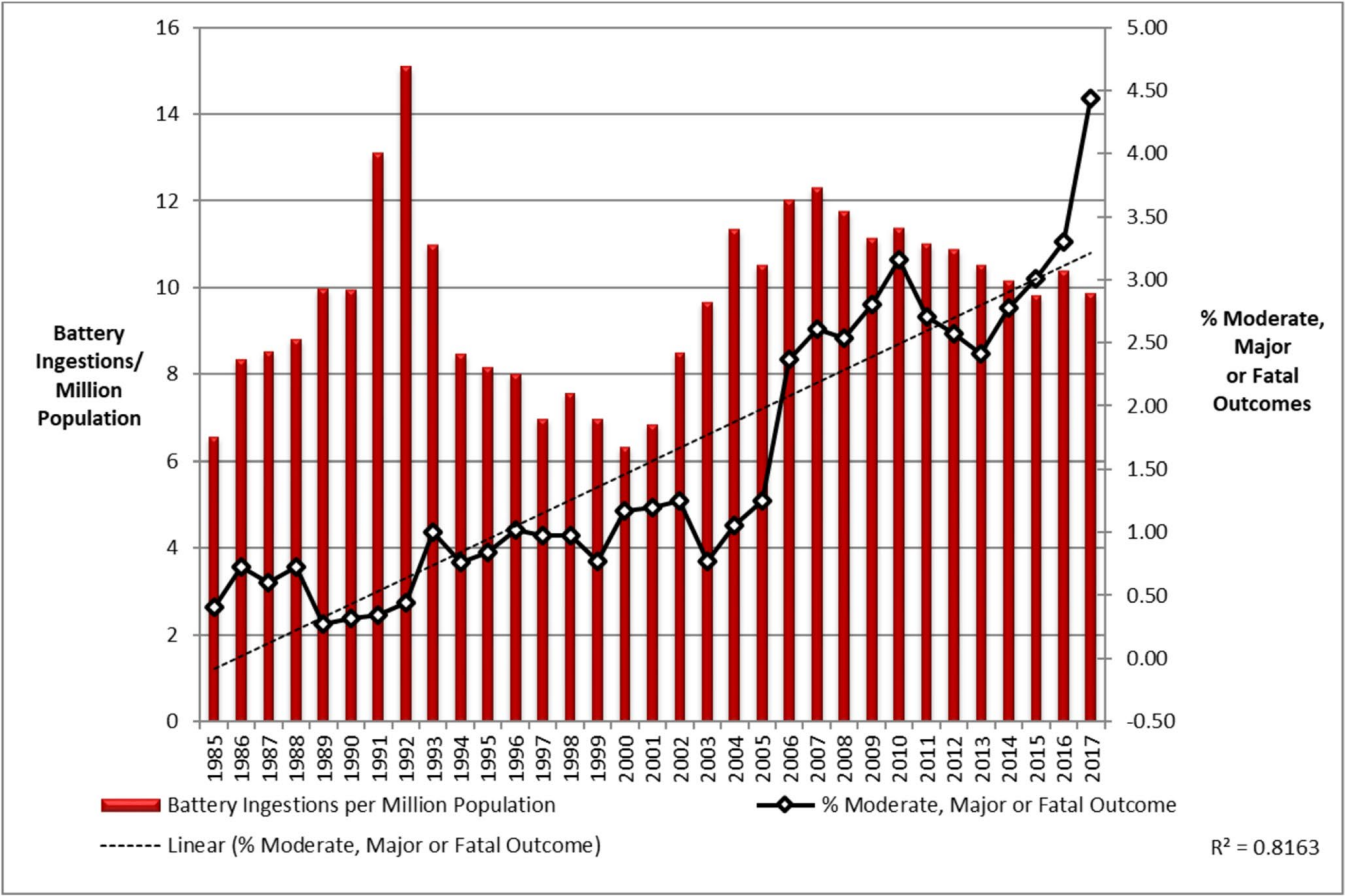
National Poison Data System (NPDS) and National Battery Ingestion Hotline (NBIH) Button Battery Ingestions Frequency and Severity for Moderate, Major, and Fatal Outcomes 1985-2017 Source: reference 11.

In addition, the GIRC Injury Data Collection App has been launched, available via the App Store and Google Store. However, due to the voluntary nature of both registries, the figures are by definition underestimated. Jatana et al. have written a paper that lends itself to extrapolation of the underestimated figures [1]. Their illustrations have been updated to 2017 by the US National Poison Data System (Fig. 1) [8]. The increase in the 1990s is largely due to the replacement of 1.5 V alkaline BBs by 3 V Lithium BBs, which led to more damage. The left vertical axis shows that in the 2010s the annual number of registered BB ingestions increased to about 10 per million inhabitants. Of this number of ingestions, approximately 0.52% of the victims died or suffered serious complications. The number of victims increased significantly until 2017. Linear extrapolation to 2025 then leads to approximately 0.72% of mutilated or deceased children.

June 12 is International Button Battery Awareness Day. This date was chosen to commemorate the birthday of Reese Hamsmith, who died at the age of 18 months after BB ingestion. On June 12, 2023, a news report from Australia (population at that time approximately 26.5 million) reported that twenty children were hospitalized each week after swallowing a BB [9]. This equates to 39 cases of BB ingestion in Australia per million inhabitants per year. Published numbers vary widely, with a minimum of 10 per million inhabitants per year.

The actual number of ingestions and their consequences can be estimated using relevant parameters. The calculations are explained in Table 1.

**Table 1:** Number of ingestions of button batteries (upper left), leading to range of number of victims with serious or fatal outcomes (lower right)

			Conversion to world population (8,1 billion)
Fact 1	Reported number of ingestions per million inhabitants per year (USA (8) and Australia (9))	10–39	81,000–315,900
Fact 2	% serious or fatal outcome (1)	Multiplication factor 0.72%	583–2,274
Fact 3	Underreporting rate of BB ingestion over 91% (6), yielding to correction factor of minimally 3	Multiplication factor 3	1,749–6,822
Fact 4	Population under the age of 15 on the African continent (11)	40%	Not applicable
Fact 5	Population under the age of 15 in North America (11)	18%	Not applicable
Fact 6	Global average percentage of children under 15 (10)	25%	Not applicable
Fact 7	Multiplication factor to correct facts 5 and 6	25/18	2,429–9,475
Estimate 1	Large correction for national health expenditure per capita, yielding a higher number of victims	Multiplication factor ≥ ≥ 1	3,000–9,500
Estimate 2	Small correction for mitigating effect of local sales of BBs, higher in Western countries	Multiplication factor ≤ 1	3,000–8,000

The world population currently stands at 8.1 billion. With 10 to 39 ingestion incidents per million people, an estimated 81,000 button batteries are ingested each year using the lowest figure. With a serious or fatal outcome of 0.72% [1], this equates to (0.0072 × 81,000 =) 583 serious or fatal outcomes from button battery ingestion per year worldwide. This is the absolute minimum confirmed number based on the US voluntary figures.

The underreporting rate of BB ingestion is 91% [6]. As a conservative estimate, this means that the estimated number of BB ingestions derived from the National Poison Data System for the US could be at least tripled. To compensate for this underreporting, the estimated number of victims worldwide would therefore increase to (3 × 583 =) 1,749 per year.

The higher the birth rate in a country, the larger the proportion of the pediatric population and the greater the probability of BB ingestion. In 2024, Niger had the highest birth rate in the world, with an estimated birth rate of 44.4 births per 1,000 (male and female) inhabitants [11]. The United States has a birth rate of 11.0 per 1,000 inhabitants. All ten countries with the highest birth rates in the world are in sub-Saharan Africa [11]. These birth rates result in 40% of people under the age of 15 on the African continent, compared to 18% in North America and 16% in Europe [11]. The global average percentage of children under the age of 15 is 25% [10]. This increases the assumed number of pediatric victims by (25 / 18 × 1,749 =) 39% to 2,429.

Rapid removal of a lodged button battery (< 2 h) leads to fewer consequences compared to longer retention [11]. Rapid removal requires good quality of health care. The higher the national health expenditure per capita, the greater the chance that a lodged battery can be removed quickly. In other words, the lower the health care expenditure, the longer the delay. According to WHO, total annual health expenditure per person in 2020 ranged from less than US$20 in Burundi, Gambia and Madagascar to more than US$10,000 in Switzerland and the United States. It seems reasonable to increase the number of (mainly) children per year who die or develop serious complications as a result of BB ingestion by an unknown factor and express the number as (2,429 x (> 1,0) =) “thousands” per year worldwide, with a conservative estimate between 3,000 and 8,000 victims per year.

However, a mitigating effect on the number of victims is the local sales of BBs. Asia-Pacific is the largest market, with a share of more than 45%, followed by North America and Europe with a share of approximately 25% and 20% [12]. Adjusting these numbers to the level of national health care involves too many assumptions and uncertainties, and is therefore unrealistic.

The authors acknowledge that their analysis relies heavily on U.S.-based and Western data. Incorporating data from non-Western regions would make our global estimates more complete and strengthen our arguments for international policy changes. However, this aspect is impossible due to the lack of national registries. BB ingestions have been reported globally as case reports, as evidenced by dozens of reports in the appendix of a 2024 review on this topic [13].

All in all, the sheer number of thousands of victims of BB ingestion each year, with major or fatal consequences, calls for stricter regulations that go beyond just safe packaging. A positive step is to expand existing government standards. This will reduce accidental access to button batteries not only in toys. From 2023, the EU will be expanding the regulations and standards (as set out in the European Regulation 2023/1542) to a wider range of household products than is currently the case. Similar measures have been taken in Australia and the United States. There are currently several attempts to design a button battery that will be safe and prevent them from having their potentially lethal effect. At least five different patent families have been filed or granted to mitigate or prevent the consequences of tissue necrosis after ingestion.

Current safety measures are either preventive or measures to stop the power supply if swallowed. Preventive measures include child-proof packaging (applied by all manufacturers), bitter taste (Duracell), or blue dye (Energizer). Measures to completely stop power supply or at least mitigate the shorting current can be achieved by utilizing technology described in patent families by either Duracell, Energizer, Fused Button Battery or Landsdowne Labs. If manufacturers were to implement and produce one of these designs for safer button batteries, and especially when required by the relevant authorities, this would be a game changer eliminating the need for time-critical management, by removing the primary cause. This would tackle the problem at its root, namely the battery itself.

The annual number of victims of swallowed BBs cannot be estimated with much precision, but at least 3,000 per year is a conservative estimate, with an uncertain upper limit, but most likely somewhere around 8,000.
